# Dehydration does not drive host behavioural manipulation by hairworms

**DOI:** 10.1371/journal.pone.0332641

**Published:** 2025-09-23

**Authors:** Louise M. Coates, Kieran Reynolds, Mara Carey-Wood, Dominika Lastik, Chris Vennard, Jean-François Doherty, Eddy Dowle, Benjamin J. Matthews, Leonard J. Foster, Stuart E. Reynolds, Vicky L. Hunt

**Affiliations:** 1 Department of Life Sciences, University of Bath, Bath, United Kingdom; 2 Department of Zoology, University of British Columbia, Vancouver, British Columbia, Canada; 3 Michael Smith Laboratories, Department of Biochemistry & Molecular Biology, University of British Columbia, Vancouver, British Columbia, Canada; 4 Department of Anatomy, University of Otago, Dunedin, New Zealand; Lusofona University of Humanities and Technologies: Universidade Lusofona de Humanidades e Tecnologias, PORTUGAL

## Abstract

Nematomorphs are parasitic worms of arthropods, which complete their life cycle via behavioural manipulation of their host so that they can enter water to find a mate. Although this behaviour is readily observed, the underlying mechanism is largely unknown; previously proposed hypotheses include an attraction to polarised light, increased erratic behaviour and dehydration-driven behaviour. Here, we investigated the ‘*Dehydration Hypothesis*’, which posits that nematomorphs either induce dehydration or mimic dehydration through biosynthetic changes to stimulate host water-seeking behaviour. House crickets, *Acheta domesticus,* were experimentally deprived of water and their behaviour compared to crickets infected with the nematomorph *Paragordius varius*. Both infected and dehydrated crickets were more likely to interact with water than uninfected, hydrated crickets. However, dehydrated crickets preferred to submerge their heads in the water compared to infected crickets which preferred to fully enter the water. Quantitative mass spectrometry of cricket haemolymph identified unique proteomic signatures of infection (27 differentially abundant proteins, infected *cf.* control) and dehydration (17 differentially abundant proteins, dehydrated *cf.* control). Our results indicate that dehydration is not a strong driving mechanism for behavioural manipulation by nematomorphs, but nevertheless infected and dehydrated share the increased tendency of dehydrated crickets to interact with water. Our data also provide new insights into the proteomic response during nematomorph infection. Notably, we observed a decrease in the cricket egg yolk protein vitellogenin and the carbohydrate digestion enzyme α-amylase, and an increase in abundance of the immune related hemocyanin protein family.

## Introduction

**Fig 1:**
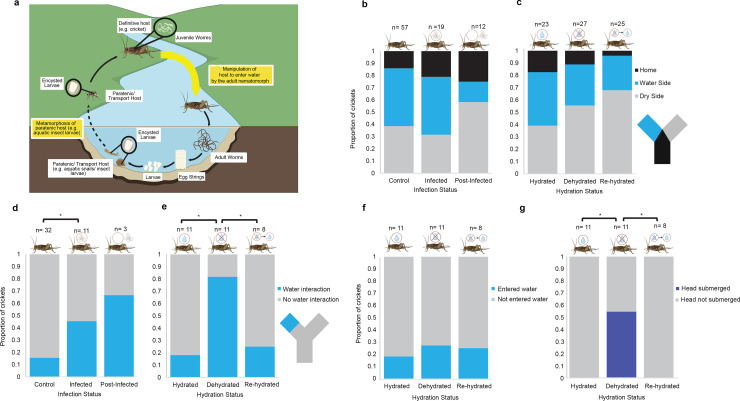
Water interaction behaviour in infected and dehydrated crickets. **(a)** Typical nematomorph life cycle. Free-living adults reproduce in the aquatic environment. Females lay egg strings, from which the larvae hatch and congregate on the waterbed.Larvae are consumed by a paratenic ‘transport’ host (e.g., aquatic insect larvae) in which the larvae encyst. The larvae are transported to the terrestrial environment during metamorphosis of the paratenic host (e.g., aquatic insect larvae), where this host is consumed by the definitive host (e.g., cricket). The nematomorph larvae excyst and develop into parasitic juveniles. Once mature, the adult nematomorphs manipulate the hosts behaviour, driving it into water for the mature worms to emerge and continue the cycle. Created by BioRender.com. Behavioural assay outcomes for the initial arm choice in the Y-maze, showing the proportion of individuals in the home arm (black), dry arm (grey) and water arm (blue) for uninfected, infected and post-infected crickets **(b)**, and hydrated, dehydrated and rehydrated crickets **(c)**. The initial choice was random and not influenced by infection or hydration status. Behaviour upon encountering the water trough, showing the proportion of individuals which interacted with the water trough (blue) or did not interact with the water trough (grey) for uninfected, infected and post-infected crickets **(d)**, and hydrated, dehydrated and rehydrated crickets **(e)**. Water interaction behaviours displayed by dehydrated crickets, with the proportion of crickets entering the water (blue) or not (grey) between hydrated, dehydrated and re-hydrated crickets **(f)**. Head submersion behaviour in dehydrated crickets (deep blue) which encountered the water trough compared to hydrated and re-hydrated crickets **(g)**. * = p < 0.05 (Post hoc comparisons using the t-test).

Parasitism has independently evolved 200 times, and almost half of all animals are considered parasites [1,2]. Many parasites manipulate their hosts’ behaviour to either complete their life cycle [3] or aid in transmission [4]. Nematomorphs, also known as hairworms, are an entirely parasitic invertebrate phylum belonging to the Ecdysozoa [2,5], which manipulate the behaviour of their definitive host to complete their life cycle (**Fig 1a**). Juvenile nematomorphs are obligate parasites of terrestrial arthropods, and once mature, the adult worms emerge as free-living in the aquatic environment via manipulation of host behaviour so that the hosts are more likely to enter water [6–9]. Although this behaviour is readily observed, the underlying mechanism used by the parasite to manipulate host behaviour is largely unknown; previously proposed hypotheses include an attraction to polarised light [9,10], increased erratic behaviour [8,11] and dehydration-driven behaviour [12,13].

The route of inducing the water entry behaviour in nematomorph-infected terrestrial arthropods via dehydration has been previously suggested but not experimentally tested [12,13]. Dehydration can alter an organisms behaviour by increasing their activity to remove themselves from dehydration-associated environments or to seek out water [14–16]), or reducing their activity to prevent greater water loss [17,18]). In arthropods responses to dehydration include reduced locomotion, relocating to more favourable conditions [19], altering their diet [20,21], or energy metabolism [22], increasing the production of metabolic water [23], or a combination of the above [24]. Additionally, dehydration can alter host-vector interactions, with implications for disease transmission. Mosquito vectors for diseases including West Nile virus, malaria, dengue and yellow fever increase their activity, host landings and host-blood feeding during dehydration, which could increase disease transmission during periods of low water availability, and may become more common due to the effects of climate change [25]. The tick vectors for tick-borne diseases (e.g., Lyme disease, tick-borne encephalitis) also have greater activity and host attachment levels during dehydration [26,27].

Dehydration has been associated with host-parasite interactions; the parasitic wasp *Pachycrepoideus vindemmiae* uses host-feeding on drosophila pupae to increase their uptake of water when dehydrated [28]. Previous work has demonstrated that taurine levels were reduced in the nematomorph-infected *Nemobius sylvestris* brain [13]. Taurine has a role in regulating brain osmoprotection, thus, it was hypothesised that nematomorphs could induce thirst in their host to encourage it to seek out water [12,13]. Two mechanisms could underlie this behaviour: direct induction of dehydration or mimicry via biosynthetic changes, such as protein alterations. Understanding the mechanisms driving changes in insect behaviour could have important implications for both pest control and invertebrate conservation strategies.

Here, we tested the *‘Dehydration Hypothesis’* of nematomorph host manipulation in the house cricket *Acheta domesticus* infected with the nematomorph parasite *Paragordius varius*. We investigated and compared (i) water-seeking and water-interacting behaviour in dehydrated and infected crickets, (ii) the proteomic signatures of dehydration and infection. Further protein differential abundance analysis identified key protein families associated with nematomorph infection.

## Results

### Infected and dehydrated crickets do not seek out water in a Y-maze

To determine if crickets infected with *P. varius* seek out water, a Y-maze choice test (water *vs.* no water) was conducted for uninfected control crickets, crickets infected with *P. varius* at 26 days post-infection (dpi), post-infected crickets at 40 dpi after the nematomorph has been released (infection experiment), hydrated control crickets, dehydrated crickets and rehydrated crickets (dehydration experiment). All crickets explored the Y-maze, but the proportion of crickets that entered either the water or dry arm at initial choice did not differ from that expected by random choice for both infection (Fisher’s exact test, *P* = 0.1601; **Fig 1b**) and hydration status (Fisher’s exact test, *P* = 0.3076; **Fig 1c**), suggesting that crickets, including infected and dehydrated crickets, did not demonstrate a statistically significant preference for the water arm from a distance of 1 m in the Y-maze arena. Behavioural assays were conducted both during the day (10 am – 6 pm) and at night (10:30 pm – 6 am) to identify potential differences associated with circadian rhythms. No difference in behaviour was found for day and night experiments and these data were therefore combined for analyses (S1**–**S4 Tables).

### Infected and dehydrated crickets are more likely to interact with water than controls

We next investigated how crickets interact with water when it is encountered, i.e., do crickets enter the water or submerge their head in the water (water interaction) or do not enter the water (no water interaction). Once crickets encountered the water, i.e., reached the edge of the trough, infected crickets (Fisher’s exact test, *P* = 0.0439, **Fig 1d**; S3 Table) and dehydrated crickets (Fisher’s exact test, *P* = 0.0063, **Fig 1e**; S4 Table) were more likely to interact with the water than uninfected and hydrated control crickets. However, the water interaction behaviour of infected and dehydrated crickets was different. Dehydrated crickets were not more likely than hydrated, uninfected crickets to fully enter the water (Fisher’s exact test, *P* > 0.9999, **Fig 1f**), but the probability of submerging their heads in the water for a prolonged period (e.g., > 5 seconds) was increased (Fisher’s exact test, *P* = 0.0016, **Fig 1g**). Head submerging behaviour was not observed in infected crickets, and all water interactions by infected crickets involved fully entering the water (S1 Video and S2 Video; examples of dehydrated and infected water interaction behaviour respectively). Time of day had no effect on the likelihood of infected or dehydrated crickets interacting with the water (S3 Table and S4 Table respectively) or on the head submerging behaviour in dehydrated crickets (Fisher’s exact test, *P* = 0.1818).

### Proteomic signatures of dehydration and infection do not overlap

Although the specific water interaction behaviour differed between infected crickets that fully entered the water and dehydrated crickets preferring to submerge their heads, dehydration did increase the probability of interaction with water and could therefore, at least in part, be involved in driving the infected cricket behaviour. We therefore sought to identify whether a molecular signature of dehydration in crickets is present within infected crickets by conducting quantitative proteomics on haemolymph samples from experimentally dehydrated and nematomorph-infected crickets.

Differential abundance analysis identified 27 significantly differentially abundant proteins in the infected crickets *cf.* control, and 17 significantly differentially abundant proteins in the dehydrated crickets *cf*. control (ANOVA q < 0.05; S5 Table) and there was no overlap between these protein sets. This indicates that the proteomic signatures of infection and dehydration are distinct. There was also no overlap between infection-associated and dehydration-associated proteins when accounting for time of day (S6 Table and S7 Table); number of significantly differentially abundant proteins for day: infected = 32 proteins; dehydrated = 19 proteins; and night: infected = 13 proteins; dehydrated = 19 proteins, indicating that the proteomic signatures of infection and dehydration are distinct.

### Infection is associated with changes in abundance of immune- and reproductive-associated proteins

**Fig 2:**
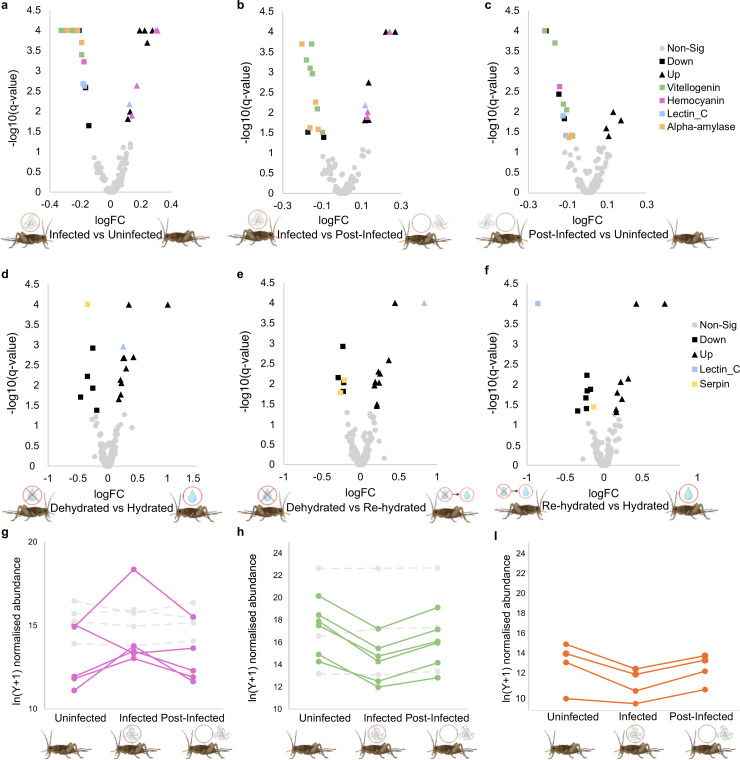
Differentially abundant proteins in the haemolymph. Volcano plots of the differentially abundant proteins in the haemolymph between infected and uninfected crickets **(a)**; infected and post-infected crickets **(b)**; post-infected and uninfected crickets **(c)**; dehydrated and hydrated crickets **(d)**; dehydrated and re-hydrated crickets **(e)**; and re-hydrated and hydrated crickets **(f)**.Proteins with non-significant differential abundance are represented by circles (grey), proteins with a significant increase in abundance in the first group are represented by triangles (black) and proteins with a significant decrease in abundance in the first group are represented by squares (black). Proteins of interest are coloured for vitellogenin (green), hemocyanin (pink), lectin_C (blue), α-amylase (orange) and serpin (yellow), with the shape corresponding to the differential abundance (i.e., square representing decreased abundance and triangles representing increased abundance). Abundance plot of the significantly differentially abundant proteins in comparison to their non-significant counterparts (grey) (n.b. only proteins detected by mass spectrometry are included) for the hemocyanins **(g)**, with significantly differentially abundant proteins in pink, the vitellogenins **(h)**, with significantly differentially abundant proteins in green, and the α-amylases **(i)**, with significantly differentially abundant proteins in orange between uninfected, infected and post-infected crickets. Significance is at the q < 0.05 level.

Overall, mass spectrometry identified 134 proteins in the uninfected, infected and post-infected cricket haemolymph (S1 Data in S1 File). Of these, 31 proteins had significant differential abundances for at least one pairwise comparison (ANOVA q < 0.05; S8 Table; **Fig 2. a-c**). Four hemocyanin proteins (out of 18 predicted in the *A. domesticus* genome), which have immune related functions [29], increased in abundance in infected crickets. These then decreased in abundance post-infection, returning to control level (**Fig 2g**). Other proteins with immune and regulatory functions with significant differential abundance include an increase in abundance of a leucine-rich repeat protein 8 (1/133 predicted in the *A. domesticus* genome), a lectin_C (1/86) and an aspartic peptidase (1/1), and a reduction in abundance of lectin_C (2/86) and a pathogenesis-related thaumatin (1/3) in infected crickets. Also reduced in abundance in infected crickets were six vitellogenin proteins (out of 12 predicted in the *A. domesticus* genome; **Fig 2h**), which are associated with roles in both reproduction and defence [30], and four α-amylases (out of 15 predicted in the *A. domesticus* genome; **Fig 2i**), which are associated with carbohydrate digestion [31]. No proteins were significantly differentially abundant in infected crickets between day and night, indicating a similar response to infection irrespective of time of day (S2 Data in S1 File).

### Dehydration is associated with an increased abundance of stress related proteins

Mass spectrometry identified 388 proteins in the haemolymph of hydrated, dehydrated and rehydrated crickets (**S3 Data** in S1 File), of which 26 were significantly differentially abundant in at least one pairwise comparison (ANOVA q < 0.05; S9 Table; **Fig 2. d-f**). Proteins with an increase in abundance in dehydrated crickets include a ribosomal protein S19 (1/1 predicted in the *A. domesticus* genome), lectin_C (2/86), which is distinct from the lectin_C proteins differentially abundant in the infected crickets above, and diverse enzymatic proteins. Proteins reduced in abundance included a carboxypeptidase D (1/4), a thioredoxin (1/35) and serpins (2/21). Within the dehydrated crickets, seven proteins increased in abundance during the day, including a lectin_C (1/86) and leucine-rich repeat proteins (3/133), and 20 proteins reduced in abundance during the day, including ribosomal proteins (2/107) and diverse enzymatic proteins (e.g., trypsin (2/142)), suggesting that the proteomic dehydration response is diurnal (**S4 Data File** in S1 File).

## Discussion

We have investigated the *‘Dehydration Hypothesis’* for behavioural manipulation of the cricket host by a nematomorph parasite (*i.e.,* is water-entering behaviour driven by the parasite by inducing or mimicking dehydration in the host?). We found that both dehydrated crickets and those infected with *P. varius*, were more likely to interact with water than control crickets. However, the interaction behaviour of crickets encountering water was qualitatively different. Similar to previous findings, nematomorph-infected crickets fully entered the water [8,9,11]. This behaviour is likely driven by the nematomorph so that the parasite can emerge from its host, enter the water and find a mate. Dehydrated crickets submerged only their head in water, a behaviour not observed in nematomorph-infected crickets, presumably an act that enables rehydration. However, dehydrated crickets did not fully enter the water any more than controls. This suggests that whether dehydrated or not, uninfected crickets avoid fully entering the water, thus reducing the risk of drowning or being attacked by aquatic predators, and that this avoidance of water-entry is overridden by the parasite in the infected individuals. Within the Y-maze, neither infected nor dehydrated crickets sought out water, suggesting that within the Y-maze the initial encounter with water is random or that other cues, unavailable in the Y-maze, are used for seeking out water such as light polarisation [9,10] or chemosensory cues [32–36]. Other possible long-range cues such as humidity gradients were not tested and controlled for in the Y-maze, though the role of long-range cues (e.g., humidity) was not previously found to be involved in the water detection by infected hosts in both field and laboratory experiments [8]. An alternative driver of water detection is the two-step hypothesis [11], which proposes a first step of erratic behaviour to increase the chance of encountering water, then a switch to the second step of entering water once it is encountered. It is yet to be determined if the mechanism of manipulation is the same for all host-nematomorph systems. For example, light polarisation may be more important for the manipulation of infected mantids which reside in trees above the water [9], than for the ground-dwelling hosts (e.g., crickets).

We used quantitative proteomics to identify the proteomic signatures of infection and dehydration in the haemolymph. There was no overlap between differentially abundant infection-associated proteins and dehydration-associated proteins, further supporting that dehydration is not involved in driving host behaviour manipulation in the *P. varius*-cricket system. Our study also provides new insights into the distinct proteomic changes that occur during both nematomorph infection and dehydration. During infection and post-infection, vitellogenin proteins were consistently downregulated. The primary role of these egg yolk precursor proteins is to provide an energy source for the developing embryo, but they have also been associated with parasite and pathogen defence [30,37]. The cricket could be reducing the production of vitellogenin to reduce reproduction and reallocate resources to defence, as has been observed previously for pathogen and parasite infections in insects [38–40]. The reduction in vitellogenin in infected crickets could benefit the parasite. Nematomorphs can inhibit ovary development and egg production in female hosts and effectively castrate male hosts [41–43], enabling them to occupy the entire host body cavity or to redirect host resources for the parasites’ benefit [41]. This presents the possibility that protein abundance changes observed in the host could be parasite driven. It is also possible that the parasitic nematomorph stages could be breaking down and consuming the host fat body, where vitellogenins are produced, as has been previously observed in insect parasites [44,45], thus reducing the vitellogenin abundance. In our study, crickets which harboured nematomorphs at dissection contained noticeably fewer eggs than their uninfected and post-infected counterparts (personal observation, LMC), consistent with previous results on nematomorph infected crickets [42,46]. Further investigation is required to uncover the driving factors and consequences of reduced vitellogenin during a nematomorph infection and the mechanisms driving these changes during infection.

Hemocyanin level increased in cricket haemolymph during infection. Hemocyanins are copper-containing proteins found in invertebrates and are commonly associated with oxygen transportation [47]. However, in insects, where the tracheal system is the main mechanism for oxygen transportations, the role of hemocyanin is less well understood. During infection hemocyanins have been associated with phenoloxidase-mediated immunity and melanin production in molluscs and crustaceans [48–52]. The prophenoloxidase system is also an important component of insect innate immunity [53] and is involved in anti-nematode response in *Drosophila* [54]. The increased levels of hemocyanin observed during nematomorph infection could therefore reflect components of the anti-parasite immune response, but further investigation is needed to elucidate their exact role.

A subset of four α-amylase proteins decreased in abundance during infection, followed by post-infection recovery to control-like levels. The α-amylases are important in carbohydrate digestion, and could reflect changes in feeding behaviour or the processing of carbohydrates during infection, as has been previously observed for insects infected with nematode parasites [55]. The parasitic juvenile stage of the nematomorph life cycle absorbs nutrients from inside the host [56–58], and changes in α-amylase production or carbohydrate digestion could represent the complex dynamics of host and parasite competing for nutrients. More research is needed to understand the consequences of nematomorph infection on host nutrient acquisition.

Previous studies on insects infected with nematomorphs have suggested that host behaviour manipulation is affected by circadian rhythm and occurs either around noon (mantids infected with *Chordodes* sp [9]) or midnight (crickets infected with *P. tricuspidatus* [8]). However, here we found no difference in either the behaviour or proteomic responses of the cricket to infection between day and night trials. Our study was conducted on laboratory reared crickets and although they were maintained on a light:dark cycle, they did not face the same restrictions on their activity, e.g., foraging for food or predator avoidance, which are important in natural conditions, and which could have affected host behavioural manipulation. Our findings may also reflect species-specific differences in the timing of host behaviour manipulation or the relatively new host-parasite relationship between *P. varius* and *A. domesticus*. *P. varius* is distributed across North, Central and South America [59,60], where its natural hosts are orthopterans; including *Gryllus sp.* and *Nemobius sp.* crickets [60] and grasshoppers [61]. Although *A. domesticus* successfully works as a host in laboratory culture [46,62,63], it is unknown if it is a host of *P. varius* in the wild. The earliest possible interaction between in *P. varius* and *A. domesticus* is in the 18^th^ century when *A. domesticus* was introduced to the Americas from ships, then through the pet trade and cricket farms [64–66] and subsequently feral populations have been observed [67].

In conclusion, our results suggest that induction or mimicry of dehydration is not a driving force for host behaviour manipulation of crickets infected with *P. varius,* as evidenced by differences in water interaction behaviour of infected and dehydrated crickets and distinct proteomic signatures of infection and dehydration in the haemolymph. However, our results do identify changes in proteomic composition of the haemolymph during infection, suggesting that egg production may be affected during infection; this requires further investigation to fully understand the consequences of this for both the host and parasite. Our findings contribute to a deeper understanding of the molecular underpinnings of parasitic behavioural manipulation, with broader implications for parasite-host ecology.

## Methods

### Acheta domesticus dehydration

Laboratory reared *A. domesticus* were housed in individual 2 litre plastic containers with a portion of egg carton provided as a shelter, in a temperature-controlled room on a 14-hour light: 10-hour dark photocycle at 30°C during the light and 25°C during the dark. Control crickets were watered (cotton dampened with distilled water) and fed (rodent pellets; SDS-diets) *ad libitum*. Dehydrated crickets had their water source removed for 48 hours prior to testing in the behavioural assay. Re-hydrated crickets had their water source removed for 48 hours, then returned 48 hours prior to testing in the behavioural assay. The level of dehydration was determined from previous trials assessing a gradient of dehydration from hydrated, 24–96 hours dehydrated, and re-hydrated. Although there was no difference in the encounter rate with the water between the groups (Fisher’s exact test, *P* = 0.565), the 48-hour dehydrated group had the greatest activity and encounter rate. Note, 48 hours was selected as a suitable duration to dehydrate crickets for because the mortality rate of dehydrated crickets was > 60% at 96 hours (S1 Fig).

### Paragordius varius infection

*P. varius* larvae, previously stored at −70°C, were thawed and approximately 300 larvae were pipetted into 24 well plates filled with 2 ml of aged tap water (at least 24 hours at room temperature). A single laboratory reared *Physella acuta* snail was added to each well and allowed to feed on the larvae for approximately 72 hours, then removed and maintained in a 12 litre tank with aged tap water. Snails were starved for 24 hours prior to exposure. Snails were fed on a diet of algae wafers (Herons) and a calcium supplement as required (Forest Aquatics) for 4 weeks post-exposure. To expose *A. domesticus*, the shell of a snail was removed, and the tissue macerated with a razor blade, then fed to a 24-hour starved laboratory reared *A. domesticus*. Each cricket was fed a single snail. Control crickets were fed an unexposed snail. Two sets of crickets were exposed on subsequent days. Crickets were housed in individual compartments for 24 hours to ingest their snail tissue, then returned to individual, labelled, 2 litre containers with a portion of an egg carton as a hiding place. Crickets were fed and watered *ad libitum* as above. Female penultimate instar crickets were used at the start of trials, which moulted to adult stage prior to behavioural trials and proteome collection. Crickets were housed in a temperature-controlled room on a 14-hour light: 10-hour dark photocycle at 30°C during the light and 25°C during the dark. At 26 days post-exposure, crickets were tested in the behavioural assay. The remaining exposed crickets, not used for experiments, were placed in water on days 27, 30, 35 and 39 post-exposure to expel their worms, then tested in the behavioural assay on day 40 post-exposure to represent a post-infected group. Of the remaining crickets tested, only 3 had worms emerge to represent a post-infected group and these emerged on days 27, 30 and 35 post-exposure. No worms emerged on day 39 post-exposure.

### Behavioural assays

A Y-maze choice test (S2 Fig) was conducted in a controlled temperature room at 25°C. The Y-maze was formed of wood with a Plexiglas lid, with each arm measuring 1 m. Each arm had a trough at the end, but only one was filled with fresh room temperature tap water, to the level of the arm (depth 4.5 cm). The arm with the water trough was alternated between crickets (i.e., control and test cricket tested then the arm switched) to avoid positional bias [8,68]. Additionally, to avoid path bias, two short Plexiglas barriers were placed on both sides of the home arm near the centre of the maze to create a channel into the centre of the maze to encourage the individual to make a choice rather than following one side of the home arm into the corresponding choice arm [69]. The test cricket was transferred to a sample pot and allowed 5 minutes to acclimatise to the test room. The cricket was then placed under this pot in the end of the home arm of the maze for a further 2 minutes to acclimatise to the maze. The pot was then lifted, and the cricket was given free rein in the maze for up to 10 minutes. The test ended after 10 minutes or if the cricket entered a trough. The initial choice at the centre of the Y-maze (i.e., water or dry arm), encounters with the water trough and final position (i.e., trough or arm) of each cricket was recorded. Each cricket was tested once, then collected into a pot and put on ice for 5 minutes prior to processing in the laboratory. The choice test was conducted during the day (10 am – 6 pm) and night (10:30 pm – 6 am). During the night tests, the observer used infrared night vision goggles (Nightfox Swift) to observe the cricket. Test crickets alternated between exposed and control, and the side with the water trough alternated between each set of exposed to control crickets. The maze was wiped with ethanol between each cricket and the water replaced if a cricket encountered it.

### Haemolymph collections

Each cricket was weighed, and its length measured. Two 1.5 ml Eppendorf tubes were weighed and placed on ice, then the cricket was positioned over one of the tubes and its head cut off into the tube [70]. The haemolymph was collected from opening into the second tube. The tubes were reweighed, then stored at −70°C until use. Exposed crickets were dissected after collections to confirm their parasitic status; exposed but uninfected, exposed and infected and post-infected.

### Proteomics

Samples were sent to the Centre for Proteome Research (University of Liverpool, UK) for protein extraction and quantification through liquid chromatography-mass spectrometry (LC-MS/MS). Briefly, lysis buffer (100 mM Ammonium Bicarbonate, 2% SDS, 1X protease inhibitors) equivalent to 8 times the sample weight was added to the sample in a bead beating tube and homogenised through 3 cycles of 30 seconds, placing on ice between each cycle. Samples were centrifuged at 10,000 x g for 10 minutes at 4°C, and the protein concentration estimated using a BCA assay with BSA standards. The volume of reagents used for protein digestion varied based on the protein concentration. The protein from each sample (36–50 µg) was diluted with 50 mM Ammonium Bicarbonate (total volume 40–200 µL). Cysteine reduction was performed by adding 11.1 mg/mL DTT solution (2.5–12.8 µL) to each sample and incubating at 60°C for 10 minutes with shaking at 450–1000 rpm. Samples were then alkylated by addition of 46.6 mg/mL iodoacetamide solution (2.5–12.1 µL) and incubating for 30 minutes in the dark. SP3 beads were added (60–86.4 ng) along with ethanol to a final concentration of 60% ethanol for the protein to bind to the beads, then mixed by pipetting and incubated for ten minutes at room temperature with shaking as above. Post incubation the sample tubes were placed on a magnetic rack for 5 minutes to settle the beads, the supernatant removed, and the beads washed six times with 80% ethanol (300–1000 µL), with the tubes placed on the magnetic rack for 5 minutes between each wash to settle the beads. Remaining traces of ethanol were removed by evaporation in a speed vac for 10 minutes, and the beads resuspended in 100 µL 100 mM Ammonium Bicarbonate by sonication for 2 minutes. In-solution digestion involved adding 0.2 µg/µL Trypsin to each sample (3.5–5 µL) and incubating at 37°C overnight with shaking at 450–1000 rpm. Post digestion, the tubes were placed on a magnetic rack for 5 minutes to settle the beads, before removing the supernatant to a fresh 0.5 mL Eppendorf tube, acidifying with 1 µL TFA and incubating at 37°C for 30 minutes followed by 30 minutes on ice. Finally, the samples were dried in a speed vac and stored at −80°C until analysis was performed. Samples were analysed using either (1) Ultimate 3000 RSLC™ nano system (Thermo Scientific, Hemel Hempstead) coupled to a QExactive™ mass spectrometer (Thermo Scientific) or (2) the Evosep One Liquid chromatography system (Evosep Biosystems) coupled to a QExactive™ HF mass spectrometer (Thermo Scientific). For method (1); samples were loaded onto the trapping column (Thermo Scientific, PepMap100, C18, 300 μm X 5 mm) using partial loop injection for 7 minutes at a flow rate of 12 μL/minute with 0.1% (v/v) TFA and the sample resolved on the analytical column (Easy-Spray C18 75 µm x 500 mm 2 µm column) using a gradient of 96.2% A (0.1% formic acid), 3.8% B (80% acetonitrile,19.9% water, 0.1% formic acid) to 50% A:50% B over 90 minutes at a flow rate of 300 nL min^-1^. For method (2); samples were diluted to 5ng/uL with 0.1% formic acid and 100 ng was loaded onto the Evotips using the standard protocol [71], and analysed using the inbuilt 15 samples per day method [72]. The data-dependent program used for data acquisition consisted of a 60,000–70,000 resolution full-scan MS scan (m/z 300–2000) (AGC set to 1e6-3e6 ions with a maximum fill time of 100–250 ms). The 5–10 most abundant peaks with charge states 2–5 were selected for MS/MS using a 30,000–35,000 resolution scan with a maximum fill time of 110–300 ms and a quad width of 2Da. To avoid repeated selection of peptides for MS2 the program used a 20 second dynamic exclusion window. All analysis was performed using the LC-MS/MS raw data files. Initial analysis was performed in the PTM module of Peaks Studio 11 and Mascot using a Uniprot database for all species with the order Orthoptera to identify PTMs (in addition to carbamidomethylation of cysteine and oxidation of methionine) which should be included in subsequent analysis steps. The frequency with which individual PTMs were detected was the criterium for inclusion of any specific PTM in subsequent searches. The search criteria at this stage were a semi specific search for Trypsin with up to three missed cleavages, a precursor mass tolerance of 10 ppm and a fragment mass tolerance of 0.01 Da. The analysis involved searching the data with the static modification of carbamidomethylation (C), in addition to the dynamic modifications of Oxidation (M), Deamidation (Q) and Deamidation (N), all of which were within the top 10 PTMs found in the PTM search performed in Peaks. Additional search criteria were a semi specific search for Trypsin with up to three missed cleavages, a precursor mass tolerance of 10 ppm and a fragment mass tolerance of 0.01 Da. False positives were controlled for by combining MASCOT with Percolator using a 1% FDR cutoff. Proteins were identified using Mascot and a nucleic acid database based on the *A. domesticus* reference genome published by Dossey *et al* [73]. Label free quantification was performed using Progenesis QI (Non-Linear Dynamics). Proteins were annotated with PFAM domains by eggnog-mapper v2.1.12 [74] using the eggnog version 5.0 database with the DIAMOND [75] --ultra-sensitive option (**S5 Data File 5** in S1 File).

### Statistical analyses

Behavioural data were analysed using Fisher’s exact tests and logistic regressions, where applicable, in Graphpad Prism 10 and R (version 4.1.0). Logistic regressions were performed for (1) the initial decision made by crickets upon reaching the centre of the maze (i.e., did the cricket initially choose the water or dry arm) and (2) the outcome upon encountering the water trough (i.e., did the cricket interact with the water (enter or submerge its head for a prolonged period of time, e.g., > 5 seconds) or not). Explanatory variables included group (e.g., control, dehydrated, re-hydrated, etc.), time of day, sub-group (to account for day tested) and side of water trough. Day post-exposure, in relation to the infected crickets, was accounted for within the group category, i.e., infected represented day 26 post-exposure and post-infected represented day 40 post-exposure. Interactions were included and subsequently removed if non-significant. Crickets which remained in the home arm of the maze were excluded from analyses. The analyses of deviance were conducted using χ^2^ tests. Significance was at the *P* < 0.05 level.

The proteomic analysis was conducted on the normalised abundance data from the output of the label free quantification from Progenesis QI for the haemolymph of dehydrated, hydrated and re-hydrated crickets, and infected, uninfected and post-infected crickets separately. Proteins were filtered by peptide count, and those with a peptide count of 1 were removed from the analysis. The normalised abundance of each protein was log transformed using the In (Y + 1) transformation. Differential abundance analyses were conducted in GraphPad Prism 10 using a Two-way ANOVA for each protein with pairwise comparisons between groups using the Benjamini, Krieger and Yekutieli method to control the false discovery rate at a Q value of 0.05. Significance was at the q < 0.05 level. Differential abundance analyses were conducted between groups for day and night separately, and also between day and night for each group. The significant proteins for day and night were then compared to determine if time of day affected the protein abundance between groups (i.e., were the same proteins increased in abundance during the day and night). The fold-change (FC) of each protein was calculated by dividing the mean protein abundance of the first group by the mean protein abundance of the second group (i.e., the fold change for infected proteins in comparison to control proteins was calculated by dividing the mean protein abundance of the infected group by the mean protein abundance of the control group). A FC of greater than 1 indicates an increase in abundance, 1 indicates no difference, and less than 1 indicates a decreased abundance in the first group in comparison to the second group.

To determine if dehydration is involved in driving infected hosts to water at a proteomic level, the significant proteins for dehydrated and infected (in comparison to their respective controls) were compared to identify if the proteomic signature of dehydration is similar to infected individuals for both day and night separately.

## Supporting information

S1 Fig Encounter rate with the water in the Y-maze from 24 to 96 hours dehydration. Proportion of crickets encountering the water (blue) in the Y-maze along a dehydration gradient from hydrated, 24–96 hours dehydrated, and re-hydrated.
(DOCX)

S2 Fig Diagram and measurements of the Y-maze behavioural assay. Diagram and measurements of the Y-maze behavioural assay, including measurements of the length and width of the arms (light grey), troughs (grey and blue), and dividers for avoiding path bias (off white).
(DOCX)

S1 Table Analysis for initial choice in the Y-maze for uninfected, infected and post-infected crickets. Logistic regression analysis for the initial arm choice in the Y-maze (i.e., will the cricket choose the side with the dry or water trough) for uninfected, infected and post-infected crickets.
(DOCX)

S2 Table Analysis for initial choice in the Y-maze for hydrated, dehydrated and rehydrated crickets. Logistic regression analysis for the initial arm choice in the Y-maze (i.e., will the cricket choose the side with the dry or water trough) for hydrated, dehydrated and rehydrated crickets.
(DOCX)

S3 Table Analysis for outcome upon encountering the water trough for uninfected, infected and post-infected crickets. Logistic regression analysis for the outcome upon encountering the water trough (i.e., will the cricket interact with the water if it encounters it) for uninfected, infected and post-infected crickets.
(DOCX)

S4 Table Analysis for outcome upon encountering the water trough for hydrated, dehydrated and rehydrated crickets. Logistic regression analysis for the outcome upon encountering the water trough (i.e., will the cricket interact with the water if it encounters it) for hydrated, dehydrated and rehydrated crickets.
(DOCX)

S5 Table Haemolymph proteins with significant differential abundances between infected and dehydrated crickets to their respective controls. Proteins identified in the haemolymph of infected and dehydrated crickets compared to their respective controls with significant differential abundances (ANOVA, with FDR at 0.05).
(DOCX)

S6 Table Haemolymph proteins with significant differential abundances during the day between infected and dehydrated crickets to their respective controls. Proteins identified in the haemolymph of infected and dehydrated crickets compared to their respective controls with significant differential abundances during the day (ANOVA, with FDR at 0.05).
(DOCX)

S7 Table Haemolymph proteins with significant differential abundances during the night between infected and dehydrated crickets to their respective controls. Proteins identified in the haemolymph of infected and dehydrated crickets compared to their respective controls with significant differential abundances during the night (ANOVA, with FDR at 0.05).
(DOCX)

S8 Table Haemolymph proteins with significant differential abundances between uninfected, infected and post-infected crickets. Proteins identified in the haemolymph of uninfected, infected and post-infected crickets with significant differential abundances for at least one pairwise comparison (ANOVA, with FDR at 0.05). Highlighted proteins from Figure 2a-c, g, h.
(DOCX)

S9 Table Haemolymph proteins with significant differential abundances between hydrated, dehydrated and rehydrated crickets. Proteins identified in the haemolymph of hydrated, dehydrated and rehydrated crickets with significant differential abundances for at least one pairwise comparison (ANOVA, with FDR at 0.05). Highlighted proteins from Figure 2d-f.
(DOCX)

S1 Video Example of the dehydrated *A. domesticus* head submerging behaviour. (MOV)

S2 Video Example of the water entry behaviour for *A. domesticus* infected with *P. varius.* (MOV)

S1 File **S1 Data.** Supplementary Data File.
(XLSX)
