# Supplementary material for: Dehydration does not drive host behavioural manipulation by hairworms
Source: PLoS One. 2025 Sep 23;20(9):e0332641. doi: 10.1371/journal.pone.0332641 (PMC12456768; doi:10.1371/journal.pone.0332641)
Supplement: S1 Fig — Proportion of crickets encountering the water (blue) in the Y-maze along a dehydration gradient from hydrated, 24–96 hours dehydrated, and re-hydrated. (DOCX) [file pone.0332641.s001.docx]

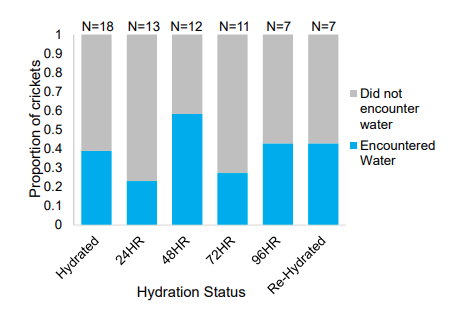


S1 Figure. Proportion of crickets encountering water (blue) in the Y-maze for hydrated, 24 to 96 hours dehydrated, and re-hydrated crickets.
