# Supplementary material for: Dehydration does not drive host behavioural manipulation by hairworms
Source: PLoS One. 2025 Sep 23;20(9):e0332641. doi: 10.1371/journal.pone.0332641 (PMC12456768; doi:10.1371/journal.pone.0332641)
Supplement: S2 Fig — Diagram and measurements of the Y-maze behavioural assay, including measurements of the length and width of the arms (light grey), troughs (grey and blue), and dividers for avoiding path bias (off white). (DOCX) [file pone.0332641.s002.docx]

**
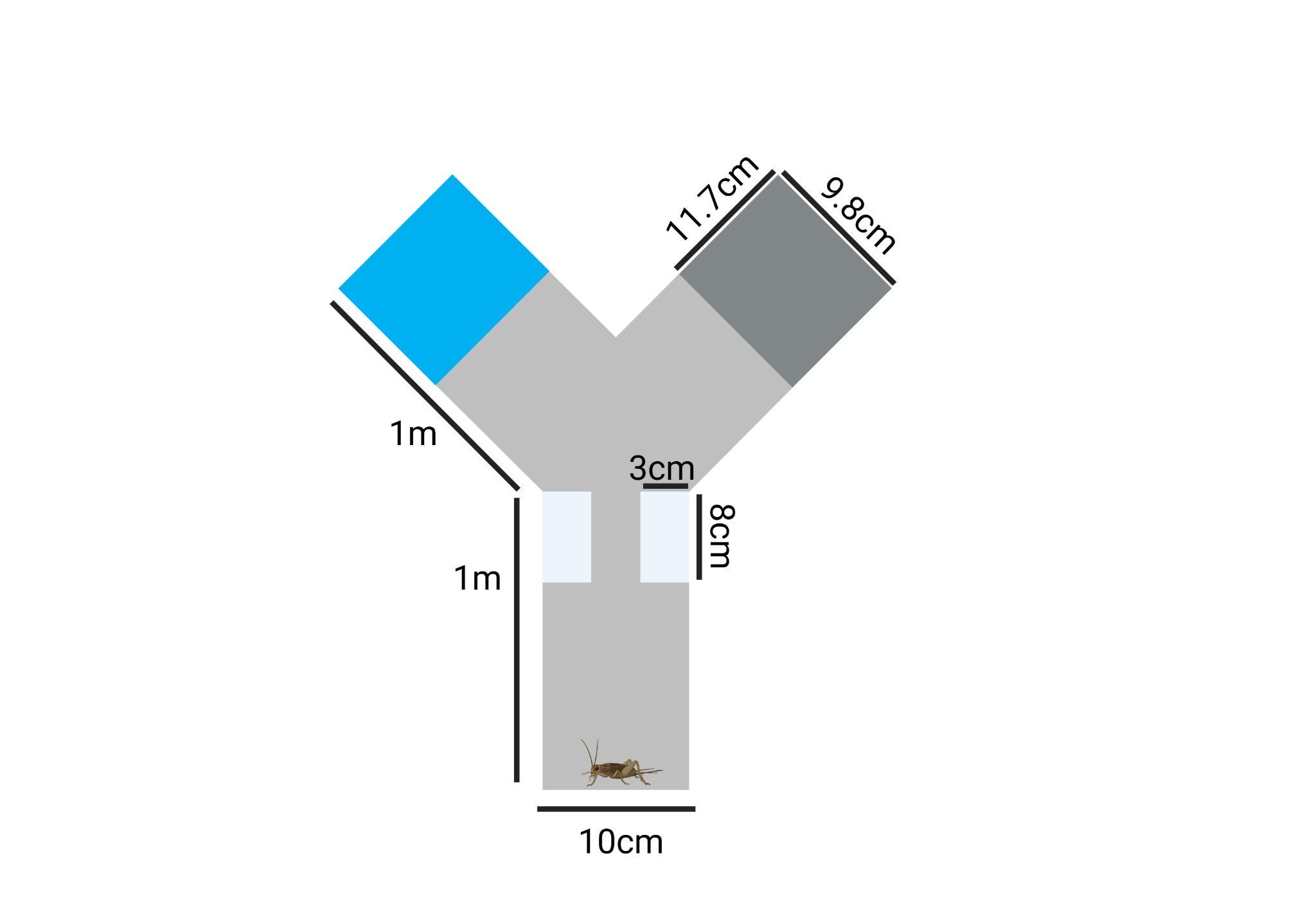
**

**S2 Figure. Diagram and measurements of the Y-maze behavioural assay, including measurements of the length and width of the arms (light grey), troughs (grey and blue), and dividers for avoiding path bias (off white).**
