# Supplementary material for: Dehydration does not drive host behavioural manipulation by hairworms
Source: PLoS One. 2025 Sep 23;20(9):e0332641. doi: 10.1371/journal.pone.0332641 (PMC12456768; doi:10.1371/journal.pone.0332641)
Supplement: S1 Table — Logistic regression analysis for the initial arm choice in the Y-maze (i.e., will the cricket choose the side with the dry or water trough) for uninfected, infected and post-infected crickets. (DOCX) [file pone.0332641.s003.docx]

**S1 Table. Logistic regression analysis for the initial arm choice in the Y-maze (i.e. will the cricket choose the side with the dry or water trough) for uninfected, infected and post-infected crickets.**

| Source | d.f | Deviance | Pr(Chi) |
| --- | --- | --- | --- |
| Group | 2 | 97.142 | 0.1399 |
| Time of day | 1 | 96.206 | 0.3333 |
| Test Day | 1 | 95.026 | 0.2773 |
| Side of Water Trough | 1 | 87.126 | 0.0049 |
| Residual | 72 | 101.076 |  |
