# Supplementary material for: Dehydration does not drive host behavioural manipulation by hairworms
Source: PLoS One. 2025 Sep 23;20(9):e0332641. doi: 10.1371/journal.pone.0332641 (PMC12456768; doi:10.1371/journal.pone.0332641)
Supplement: S2 Table — Logistic regression analysis for the initial arm choice in the Y-maze (i.e., will the cricket choose the side with the dry or water trough) for hydrated, dehydrated and rehydrated crickets. (DOCX) [file pone.0332641.s004.docx]

**S2 Table. Logistic regression analysis for the initial arm choice in the Y-maze (i.e. will the cricket choose the side with the dry or water trough) for hydrated, dehydrated and rehydrated crickets.**

| Source | d.f | Deviance | Pr(Chi) |
| --- | --- | --- | --- |
| Group | 2 | 2.4798 | 0.2896 |
| Time of day | 1 | 0.3831 | 0.5359 |
| Test Day | 1 | 0.0001 | 0.9945 |
| Side of Water Trough | 1 | 0.7485 | 0.3870 |
| Residual | 66 | 89.495 |  |
