# Supplementary material for: Dehydration does not drive host behavioural manipulation by hairworms
Source: PLoS One. 2025 Sep 23;20(9):e0332641. doi: 10.1371/journal.pone.0332641 (PMC12456768; doi:10.1371/journal.pone.0332641)
Supplement: S3 Table — Logistic regression analysis for the outcome upon encountering the water trough (i.e., will the cricket interact with the water if it encounters it) for uninfected, infected and post-infected crickets. (DOCX) [file pone.0332641.s005.docx]

**S3 Table. Logistic regression analysis for the outcome upon encountering the water trough (i.e. will the cricket interact with the water if it encounters it) for uninfected, infected and post-infected crickets.**

| Source | d.f | Deviance | Pr(Chi) |
| --- | --- | --- | --- |
| Group | 2 | 6.0899 | 0.0476 |
| Time of day | 1 | 0.2288 | 0.6324 |
| Test Day | 1 | 3.5797 | 0.0585 |
| Side of Water Trough | 1 | 1.1780 | 0.2778 |
| Residual | 45 | 52.805 |  |
