# Supplementary material for: Dehydration does not drive host behavioural manipulation by hairworms
Source: PLoS One. 2025 Sep 23;20(9):e0332641. doi: 10.1371/journal.pone.0332641 (PMC12456768; doi:10.1371/journal.pone.0332641)
Supplement: S4 Table — Logistic regression analysis for the outcome upon encountering the water trough (i.e., will the cricket interact with the water if it encounters it) for hydrated, dehydrated and rehydrated crickets. (DOCX) [file pone.0332641.s006.docx]

**S4 Table. Logistic regression analysis for the outcome upon encountering the water trough (i.e. will the cricket interact with the water if it encounters it) for hydrated, dehydrated and rehydrated crickets.**

| Source | d.f | Deviance | Pr(Chi) |
| --- | --- | --- | --- |
| Group | 2 | 11.1944 | 0.0037 |
| Time of day | 1 | 1.2457 | 0.2644 |
| Test Day | 1 | 2.8445 | 0.0917 |
| Side of Water Trough | 1 | 1.1327 | 0.2872 |
| Residual | 29 | 41.054 |  |
