# Supplementary material for: Dehydration does not drive host behavioural manipulation by hairworms
Source: PLoS One. 2025 Sep 23;20(9):e0332641. doi: 10.1371/journal.pone.0332641 (PMC12456768; doi:10.1371/journal.pone.0332641)
Supplement: S5 Table — Proteins identified in the haemolymph of infected and dehydrated crickets compared to their respective controls with significant differential abundances (ANOVA, with FDR at 0.05). (DOCX) [file pone.0332641.s007.docx]

**S5 Table. Proteins identified in the haemolymph of infected and dehydrated crickets compared to their respective controls with significant differential abundances (ANOVA, q < 0.05).**

| **Protein (*A. domesticus* annotation name)** | **q value** | **FC** | **DE** |
| --- | --- | --- | --- |
| **Infected vs. Uninfected** | | | |
| 𝛼-amylase (ANN04619) | 0.0002 | 0.87674 | DOWN |
| 𝛼-amylase (ANN09857) | <0.0001 | 0.861268 | DOWN |
| 𝛼-amylase (ANN11861) | <0.0001 | 0.821104 | DOWN |
| Aspartic peptidase (ANN14595) | <0.0001 | 1.142958 | UP |
| Epidermal growth factor-like protein 7 (ANN03781) | 0.0154 | 1.082506 | UP |
| FK506-binding protein (ANN09605) | 0.0102 | 1.093624 | UP |
| Glucose-methanol-choline oxidoreductase (ANN04429) | 0.0026 | 0.892396 | DOWN |
| Glycosyl hydrolase 9 (ANN06870; ANN06872; ANN27072; ANN06874; ANN06871) | 0.0228 | 0.9059 | DOWN |
| Hemocyanin (ANN12312) | 0.0128 | 1.102369 | UP |
| Hemocyanin (ANN12313) | 0.0023 | 1.128141 | UP |
| Hemocyanin (ANN12315; ANN06621) | <0.0001 | 1.239424 | UP |
| Hemocyanin (ANN17126) | 0.0006 | 0.885714 | DOWN |
| Hemocyanin (ANN20571; ANN20570; ANN20572; ANN00593) | <0.0001 | 1.230718 | UP |
| Lectin_C (ANN07674) | 0.0024 | 0.887906 | DOWN |
| Lectin_C (ANN18965) | 0.0021 | 0.882902 | DOWN |
| Lectin_C (ANN19004) | 0.0066 | 1.09009 | UP |
| Leucine-rich repeat 8 (ANN22820) | <0.0001 | 1.169014 | UP |
| Pathogenesis-related thaumatin (ANN19136) | <0.0001 | 0.848138 | DOWN |
| Protein of unknown function (ANN07851) | 0.0002 | 1.182617 | UP |
| Protein of unknown function (ANN12403) | <0.0001 | 1.211335 | UP |
| Reverse transcriptase (ANN02367) | <0.0001 | 0.866347 | DOWN |
| Vitellogenin (ANN00056) | <0.0001 | 0.853671 | DOWN |
| Vitellogenin (ANN00057) | <0.0001 | 0.797765 | DOWN |
| Vitellogenin (ANN00579) | 0.0004 | 0.876664 | DOWN |
| Vitellogenin (ANN00622) | <0.0001 | 0.803488 | DOWN |
| Vitellogenin (ANN20361) | <0.0001 | 0.842466 | DOWN |
| Vitellogenin (ANN20363) | <0.0001 | 0.837486 | DOWN |
| **Dehydrated vs. Hydrated** | | | |
| 40S Ribosomal protein S19 (ANN09419) | 0.0038 | 1.238688 | UP |
| Aldehyde dehydrogenase (ANN16559) | <0.0001 | 2.012444 | UP |
| Bifunctional purine biosynthesis protein (ANN06056) | 0.0117 | 0.841283 | DOWN |
| CAP (cysteine-rich secretory proteins, antigen 5, pathogenesis-related 1) protein (ANN23010) | 0.0001 | 1.279921 | UP |
| Carboxypeptidase D (ANN23320; ANN15569) | 0.0196 | 0.729955 | DOWN |
| ELFV Dehydrogenase (ANN10405) | 0.002 | 1.349766 | UP |
| Enoyl-CoA hydratase (ANN11765) | 0.0073 | 1.162069 | UP |
| Glycerol-3-phosphate dehydrogenase NAD (ANN06122) | 0.0168 | 1.163904 | UP |
| IIV6 (CIV) dUTPase-like protein (ANN28560; ANN28248; ANN28638; ANN28955; ANN28981; ANN28919; ANN28747; ANN28591) | 0.0219 | 1.137481 | UP |
| Lectin_C (ANN18972; ANN18963; ANN18964) | 0.0011 | 1.19967 | UP |
| Mitochondrial ATP synthase D (ANN05233) | 0.0088 | 1.172743 | UP |
| Protein of unknown function (ANN10400) | 0.0415 | 0.881481 | DOWN |
| Protein of unknown function (ANN11661) | 0.0021 | 1.21365 | UP |
| Protein of unknown function (ANN14409) | 0.0012 | 0.840683 | DOWN |
| Ribophorin_II (ANN10919) | 0.0021 | 1.198712 | UP |
| Serpin (ANN13713; ANN11538) | <0.0001 | 0.790274 | DOWN |
| Thioredoxin (ANN17254) | 0.006 | 0.790782 | DOWN |
