# Supplementary material for: Dehydration does not drive host behavioural manipulation by hairworms
Source: PLoS One. 2025 Sep 23;20(9):e0332641. doi: 10.1371/journal.pone.0332641 (PMC12456768; doi:10.1371/journal.pone.0332641)
Supplement: S6 Table — Proteins identified in the haemolymph of infected and dehydrated crickets compared to their respective controls with significant differential abundances during the day (ANOVA, with FDR at 0.05). (DOCX) [file pone.0332641.s008.docx]

**S6 Table. Proteins identified in the haemolymph of infected and dehydrated crickets compared to their respective controls with significant differential abundances during the day (ANOVA, q < 0.05).**

| **Protein (*A. domesticus* annotation name)** | **q value** | **FC** | **DE** |
| --- | --- | --- | --- |
| **Infected vs. Uninfected** | | | |
| 𝛼-amylase (ANN04619) | 0.0002 | 0.853723 | DOWN |
| 𝛼-amylase (ANN09857) | <0.0001 | 0.802956 | DOWN |
| 𝛼-amylase (ANN11861) | <0.0001 | 0.773006 | DOWN |
| Aspartic peptidase (ANN14595) | 0.0028 | 1.131078 | UP |
| Coesterase (ANN16377) | 0.0006 | 0.875077 | DOWN |
| EB module (ANN23753) | 0.0182 | 1.115821 | UP |
| Glucose-methanol-choline oxidoreductase (ANN04429) | <0.0001 | 0.811184 | DOWN |
| Glycosyl hydrolase 9 (ANN06870; ANN06872; ANN27072; ANN06874; ANN06871) | 0.007 | 0.870356 | DOWN |
| Heat shock protein 20 (ANN19356; ANN19354) | 0.002 | 1.16 | UP |
| Hemocyanin (ANN12315; ANN06621) | 0.0002 | 1.200935 | UP |
| Hemocyanin (ANN17126) | <0.0001 | 0.826623 | DOWN |
| Hemocyanin (ANN20571; ANN20570; ANN20572; ANN00593) | <0.0001 | 1.197729 | UP |
| Lectin_C (ANN14210) | 0.0033 | 0.880886 | DOWN |
| Lectin_C (ANN18965) | 0.0188 | 0.891738 | DOWN |
| Lectin_C (ANN19004) | 0.0025 | 1.124736 | UP |
| Leucine-rich repeat 8 (ANN22820) | <0.0001 | 1.222727 | UP |
| Pathogenesis-related thaumatin (ANN19136) | <0.0001 | 0.764256 | DOWN |
| Peptidase M14 (ANN12865) | 0.0003 | 0.832547 | DOWN |
| Protein of unknown function (ANN07851) | 0.0018 | 1.185891 | UP |
| Protein of unknown function (ANN12403) | <0.0001 | 1.326861 | UP |
| Protein of unknown function (ANN16875) | 0.0006 | 1.113507 | UP |
| Protein of unknown function (ANN21809) | 0.0214 | 1.098872 | UP |
| Protein of unknown function (ANN21810) | 0.0141 | 1.111207 | UP |
| Protein of unknown function (ANN22454; ANN21790) | 0.0304 | 1.10007 | UP |
| Reverse transcriptase (ANN02367) | <0.0001 | 0.836376 | DOWN |
| Trehalase (ANN16573; ANN00160) | 0.0022 | 0.877468 | DOWN |
| Vitellogenin (ANN00056) | <0.0001 | 0.869369 | DOWN |
| Vitellogenin (ANN00057) | <0.0001 | 0.770166 | DOWN |
| Vitellogenin (ANN00579) | <0.0001 | 0.842917 | DOWN |
| Vitellogenin (ANN00622) | <0.0001 | 0.79773 | DOWN |
| Vitellogenin (ANN20361) | <0.0001 | 0.866512 | DOWN |
| Vitellogenin (ANN20363) | <0.0001 | 0.869184 | DOWN |
| **Dehydrated vs. Hydrated** | | | |
| 40S Ribosomal protein S19 (ANN09419) | 0.0074 | 1.287813 | UP |
| ABC transporter (ANN15718; ANN15709) | 0.0325 | 0.77387 | DOWN |
| Aldehyde dehydrogenase (ANN16559) | <0.0001 | 1.900833 | UP |
| Bifunctional purine biosynthesis protein (ANN06056) | <0.0001 | 0.64905 | DOWN |
| CAP (cysteine-rich secretory proteins, antigen 5, pathogenesis-related 1) protein (ANN23010) | <0.0001 | 2.029936 | UP |
| Carboxypeptidase D (ANN23320; ANN15569) | 0.0404 | 0.599878 | DOWN |
| ELFV Dehydrogenase (ANN10405) | 0.0402 | 0.680422 | DOWN |
| Fibrillarin (ANN16121) | <0.0001 | -3.3E-15 | DOWN |
| Leucine-rich repeat 8 (ANN08713) | <0.0001 | 1.673056 | UP |
| Leucine-rich repeat 8 (ANN24069) | 0.0245 | 1.149695 | UP |
| Lysine--tRNA ligase (ANN24110) | <0.0001 | 0.669237 | DOWN |
| Mitochondrial ATP synthase D (ANN05233) | 0.032 | 1.220307 | UP |
| Peptidase family C1 (ANN17613) | <0.0001 | 1.620189 | UP |
| Protein of unknown function (ANN11661) | 0.0104 | 1.261252 | UP |
| RHO protein GDP dissociation inhibitor (ANN18226) | 0.0002 | 1.368871 | UP |
| Ribosomal protein L28e (ANN17397) | 0.0055 | 0.765314 | DOWN |
| Serpin (ANN13863; ANN27892) | 0.0431 | 0.792425 | DOWN |
| Thiolase (ANN06329) | 0.0235 | 0.832289 | DOWN |
| tRNA synthetase (ANN17552) | <0.0001 | 0.616969 | DOWN |
