# Supplementary material for: Dehydration does not drive host behavioural manipulation by hairworms
Source: PLoS One. 2025 Sep 23;20(9):e0332641. doi: 10.1371/journal.pone.0332641 (PMC12456768; doi:10.1371/journal.pone.0332641)
Supplement: S7 Table — Proteins identified in the haemolymph of infected and dehydrated crickets compared to their respective controls with significant differential abundances during the night (ANOVA, with FDR at 0.05). (DOCX) [file pone.0332641.s009.docx]

**S7 Table. Proteins identified in the haemolymph of infected and dehydrated crickets compared to their respective controls with significant differential abundances during the night (ANOVA, q < 0.05).**

| **Protein (*A. domesticus* annotation name)** | **q value** | **FC** | **DE** |
| --- | --- | --- | --- |
| **Infected vs. Uninfected** | | | |
| Aspartic peptidase (ANN14595) | 0.012 | 1.154119 | UP |
| Calcium-binding epidermal growth factor (ANN08434) | 0.0083 | 1.202328 | UP |
| Hemocyanin (ANN12312) | 0.004 | 1.173732 | UP |
| Hemocyanin (ANN12313) | 0.0038 | 1.178511 | UP |
| Hemocyanin (ANN12315; ANN06621) | <0.0001 | 1.275174 | UP |
| Hemocyanin (ANN20571; ANN20570; ANN20572; ANN00593) | <0.0001 | 1.264151 | UP |
| Leucine-rich repeat 8 (ANN22820) | 0.0423 | 1.116836 | UP |
| Peptidase M14 (ANN12865) | 0.007 | 1.227723 | UP |
| Vitellogenin (ANN00056) | <0.0001 | 0.837758 | DOWN |
| Vitellogenin (ANN00057) | <0.0001 | 0.826554 | DOWN |
| Vitellogenin (ANN00622) | 0.0003 | 0.809299 | DOWN |
| Vitellogenin (ANN20361) | 0.0001 | 0.819764 | DOWN |
| Vitellogenin (ANN20363) | <0.0001 | 0.806434 | DOWN |
| **Dehydrated vs. Hydrated** | | | |
| Aldehyde dehydrogenase (ANN16559) | <0.0001 | 2.1137 | UP |
| Aminotransferase 5 (ANN06161) | <0.0001 | 1.508269 | UP |
| Carboxypeptidase D (ANN23320; ANN15569) | 0.0296 | 0.805125 | DOWN |
| Dehydratase (ANN22969; ANN26629) | <0.0001 | 2.723482 | UP |
| ELFV Dehydrogenase (ANN10405) | <0.0001 | 2.011254 | UP |
| Enoyl-CoA hydratase (ANN11765) | 0.0025 | 1.263726 | UP |
| Fasciclin (ANN06522) | 0.0341 | 1.202569 | UP |
| Fibrillarin (ANN16121) | 0.0007 | 1.383624 | UP |
| Fibronectin type III phosphatase (ANN25842) | 0.0481 | 1.165517 | UP |
| Glycerol-3-phosphate dehydrogenase NAD (ANN06122) | 0.0036 | 1.249284 | UP |
| IIV6 (CIV) dUTPase-like protein (ANN28560; ANN28248; ANN28638; ANN28955; ANN28981; ANN28919; ANN28747; ANN28591) | 0.0043 | 1.203276 | UP |
| Lectin_C (ANN18972; ANN18963; ANN18964) | 0.0011 | 1.294351 | UP |
| Leucine-rich repeat 8 (ANN24069) | <0.0001 | 0.58292 | DOWN |
| Lysine--tRNA ligase (ANN24110) | <0.0001 | 1.491443 | UP |
| Peptidase family C1 (ANN17613) | 0.0032 | 0.76112 | DOWN |
| Protein of unknown function (ANN10400) | 0.0483 | 0.837075 | DOWN |
| Protein of unknown function (ANN14409) | <0.0001 | 0.736188 | DOWN |
| Serpin (ANN13713; ANN11538) | 0.0002 | 0.724219 | DOWN |
| Thioredoxin (ANN17254) | 0.0003 | 0.696878 | DOWN |
