# Supplementary material for: Dehydration does not drive host behavioural manipulation by hairworms
Source: PLoS One. 2025 Sep 23;20(9):e0332641. doi: 10.1371/journal.pone.0332641 (PMC12456768; doi:10.1371/journal.pone.0332641)
Supplement: S8 Table — Proteins identified in the haemolymph of uninfected, infected and post-infected crickets with significant differential abundances for at least one pairwise comparison (ANOVA, with FDR at 0.05). Highlighted proteins from Figure 2a-c, g, h. (DOCX) [file pone.0332641.s010.docx]

**S8 Table. Proteins identified in the haemolymph of uninfected, infected and post-infected crickets with significant differential abundances for at least one pairwise comparison (ANOVA, q < 0.05). Highlighted proteins from Figure 2a-c, g, h.**

| **Protein (*A. domesticus* annotation name)** | **Comparison** | **q value** | **FC** | **DE** |
| --- | --- | --- | --- | --- |
| 𝛼-amylase (ANN04619) | Infected vs. Post-Infected | 0.0055 | 0.911785 | DOWN |
|  | Infected vs. Uninfected | 0.0002 | 0.87674 | DOWN |
| 𝛼-amylase (ANN09857) | Infected vs. Post-Infected | 0.026 | 0.920188 | DOWN |
|  | Infected vs. Uninfected | <0.0001 | 0.861268 | DOWN |
|  | Post-Infected vs. Uninfected | 0.0437 | 0.93597 | DOWN |
| 𝛼-amylase (ANN11861) | Infected vs. Post-Infected | 0.0002 | 0.869726 | DOWN |
|  | Infected vs. Uninfected | <0.0001 | 0.821104 | DOWN |
|  | Post-Infected vs. Uninfected | 0.0381 | 0.944095 | DOWN |
| 𝛼-amylase (ANN13348) | Infected vs. Post-Infected | 0.0237 | 0.89385 | DOWN |
| Aspartic peptidase (ANN14595) | Infected vs. Post-Infected | 0.0018 | 1.098639 | UP |
|  | Infected vs. Uninfected | <0.0001 | 1.142958 | UP |
| Epidermal growth factor-like protein 7 (ANN03781) | Infected vs. Post-Infected | 0.0154 | 1.084992 | UP |
|  | Infected vs. Uninfected | 0.0154 | 1.082506 | UP |
| FK506-binding protein (ANN09605) | Infected vs. Uninfected | 0.0102 | 1.093624 | UP |
|  | Post-Infected vs. Uninfected | 0.0102 | 1.094431 | UP |
| Glucose-methanol-choline oxidoreductase (ANN04429) | Infected vs. Uninfected | 0.0026 | 0.892396 | DOWN |
|  | Post-Infected vs. Uninfected | 0.0149 | 0.921808 | DOWN |
| Glycosyl hydrolase 9 (ANN06870; ANN06872; ANN27072; ANN06874; ANN06871) | Infected vs. Uninfected | 0.0228 | 0.9059 | DOWN |
| Heat shock protein 20 (ANN19356; ANN19354) | Post-Infected vs. Uninfected | 0.0163 | 1.123944 | UP |
| Hemocyanin (ANN12312) | Infected vs. Post-Infected | 0.0128 | 1.094039 | UP |
|  | Infected vs. Uninfected | 0.0128 | 1.102369 | UP |
| Hemocyanin (ANN12313) | Infected vs. Post-Infected | 0.0096 | 1.096013 | UP |
|  | Infected vs. Uninfected | 0.0023 | 1.128141 | UP |
| Hemocyanin (ANN12315; ANN06621) | Infected vs. Post-Infected | <0.0001 | 1.18299 | UP |
|  | Infected vs. Uninfected | <0.0001 | 1.239424 | UP |
| Hemocyanin (ANN17126) | Infected vs. Uninfected | 0.0006 | 0.885714 | DOWN |
|  | Post-Infected vs. Uninfected | 0.0024 | 0.906312 | DOWN |
| Hemocyanin (ANN20571; ANN20570; ANN20572; ANN00593) | Infected vs. Post-Infected | <0.0001 | 1.183108 | UP |
|  | Infected vs. Uninfected | <0.0001 | 1.230718 | UP |
| Lectin_C (ANN07674) | Infected vs. Uninfected | 0.0024 | 0.887906 | DOWN |
|  | Post-Infected vs. Uninfected | 0.0127 | 0.917404 | DOWN |
| Lectin_C (ANN18965) | Infected vs. Uninfected | 0.0021 | 0.882902 | DOWN |
|  | Post-Infected vs. Uninfected | 0.0389 | 0.926006 | DOWN |
| Lectin_C (ANN19004) | Infected vs. Post-Infected | 0.0066 | 1.085576 | UP |
|  | Infected vs. Uninfected | 0.0066 | 1.09009 | UP |
| Leucine-rich repeat 8 (ANN22820) | Infected vs. Post-Infected | <0.0001 | 1.20658 | UP |
|  | Infected vs. Uninfected | <0.0001 | 1.169014 | UP |
| Myosin (ANN17471) | Infected vs. Post-Infected | 0.0308 | 0.887407 | DOWN |
| Pathogenesis-related thaumatin (ANN19136) | Infected vs. Post-Infected | 0.0409 | 0.938193 | DOWN |
|  | Infected vs. Uninfected | <0.0001 | 0.848138 | DOWN |
|  | Pos t-Infected vs. Uninfected | 0.0037 | 0.904011 | DOWN |
| Protein of unknown function (ANN07851) | Infected vs. Post-Infected | 0.0151 | 1.098911 | UP |
|  | Infected vs. Uninfected | 0.0002 | 1.182617 | UP |
|  | Post-Infected vs. Uninfected | 0.0401 | 1.076172 | UP |
| Protein of unknown function (ANN12403) | Infected vs. Post-Infected | 0.0001 | 1.167593 | UP |
|  | Infected vs. Uninfected | <0.0001 | 1.211335 | UP |
| Protein of unknown function (ANN16875) | Post-Infected vs. Uninfected | 0.0257 | 1.068083 | UP |
| Reverse transcriptase (ANN02367) | Infected vs. Uninfected | <0.0001 | 0.866347 | DOWN |
|  | Post-Infected vs. Uninfected | <0.0001 | 0.864291 | DOWN |
| Vitellogenin (ANN00056) | Infected vs. Post-Infected | 0.0002 | 0.899634 | DOWN |
|  | Infected vs. Uninfected | <0.0001 | 0.853671 | DOWN |
|  | Post-Infected vs. Uninfected | 0.0401 | 0.948909 | DOWN |
| Vitellogenin (ANN00057) | Infected vs. Post-Infected | 0.0008 | 0.894737 | DOWN |
|  | Infected vs. Uninfected | <0.0001 | 0.797765 | DOWN |
|  | Post-Infected vs. Uninfected | 0.0002 | 0.89162 | DOWN |
| Vitellogenin (ANN00579) | Infected vs. Post-Infected | 0.0005 | 0.882851 | DOWN |
|  | Infected vs. Uninfected | 0.0004 | 0.876664 | DOWN |
| Vitellogenin (ANN00622) | Infected vs. Post-Infected | 0.0311 | 0.933749 | DOWN |
|  | Infected vs. Uninfected | <0.0001 | 0.803488 | DOWN |
|  | Post-Infected vs. Uninfected | <0.0001 | 0.860496 | DOWN |
| Vitellogenin (ANN20361) | Infected vs. Post-Infected | 0.0081 | 0.91734 | DOWN |
|  | Infected vs. Uninfected | <0.0001 | 0.842466 | DOWN |
|  | Post-Infected vs. Uninfected | 0.0065 | 0.918379 | DOWN |
| Vitellogenin (ANN20363) | Infected vs. Post-Infected | 0.0011 | 0.901458 | DOWN |
|  | Infected vs. Uninfected | <0.0001 | 0.837486 | DOWN |
|  | Post-Infected vs. Uninfected | 0.009 | 0.929036 | DOWN |
