# Supplementary material for: Dehydration does not drive host behavioural manipulation by hairworms
Source: PLoS One. 2025 Sep 23;20(9):e0332641. doi: 10.1371/journal.pone.0332641 (PMC12456768; doi:10.1371/journal.pone.0332641)
Supplement: S9 Table — Proteins identified in the haemolymph of hydrated, dehydrated and rehydrated crickets with significant differential abundances for at least one pairwise comparison (ANOVA, with FDR at 0.05). Highlighted proteins from Figure 2d-f. (DOCX) [file pone.0332641.s011.docx]

**S9 Table. Proteins identified in the haemolymph of hydrated, dehydrated and rehydrated crickets with significant differential abundances for at least one pairwise comparison (ANOVA, q < 0.05). Highlighted proteins from Figure 2d-f.**

| **Protein (*A. domesticus* annotation name)** | **Comparison** | **q value** | **FC** | **DE** |
| --- | --- | --- | --- | --- |
| 40S Ribosomal protein S19 (ANN09419) | Dehydrated vs. Rehydrated | 0.0056 | 1.194064 | UP |
|  | Dehydrated vs. Hydrated | 0.0038 | 1.238688 | UP |
| Aldehyde dehydrogenase (ANN16559) | Dehydrated vs. Rehydrated | 0.0355 | 1.159274 | UP |
|  | Dehydrated vs. Hydrated | <0.0001 | 2.012444 | UP |
|  | Rehydrated vs. Hydrated | <0.0001 | 1.735952 | UP |
| Bifunctional purine biosynthesis protein (ANN06056) | Dehydrated vs. Hydrated | 0.0117 | 0.841283 | DOWN |
| CAP (cysteine-rich secretory proteins, antigen 5, pathogenesis-related 1) protein (ANN23010) | Dehydrated vs. Hydrated | 0.0001 | 1.279921 | UP |
|  | Rehydrated vs. Hydrated | <0.0001 | 1.342223 | UP |
| Carboxypeptidase D (ANN23320; ANN15569) | Dehydrated vs. Hydrated | 0.0196 | 0.729955 | DOWN |
|  | Rehydrated vs. Hydrated | 0.045 | 0.789717 | DOWN |
| ELFV Dehydrogenase (ANN10405) | Dehydrated vs. Rehydrated | 0.0026 | 1.291325 | UP |
|  | Dehydrated vs. Hydrated | 0.002 | 1.349766 | UP |
| Enoyl-CoA hydratase (ANN11765) | Dehydrated vs. Hydrated | 0.0073 | 1.162069 | UP |
|  | Rehydrated vs. Hydrated | 0.0158 | 1.130172 | UP |
| Glycerol-3-phosphate dehydrogenase NAD (ANN06122) | Dehydrated vs. Rehydrated | <0.0001 | 1.367373 | UP |
|  | Dehydrated vs. Hydrated | 0.0168 | 1.163904 | UP |
|  | Rehydrated vs. Hydrated | 0.0214 | 0.851197 | DOWN |
| IIV6 (CIV) dUTPase-like protein (ANN28560; ANN28248; ANN28638; ANN28955; ANN28981; ANN28919; ANN28747; ANN28591) | Dehydrated vs. Hydrated | 0.0219 | 1.137481 | UP |
| Lectin_C (ANN11685) | Dehydrated vs. Rehydrated | <0.0001 | 1.781233 | UP |
|  | Rehydrated vs. Hydrated | <0.0001 | 0.551107 | DOWN |
| Lectin_C (ANN18972; ANN18963; ANN18964) | Dehydrated vs. Hydrated | 0.0011 | 1.19967 | UP |
| Lysine--tRNA ligase (ANN24110) | Dehydrated vs. Rehydrated | 0.0094 | 1.183824 | UP |
|  | Rehydrated vs. Hydrated | 0.0143 | 0.860759 | DOWN |
| Mitochondrial ATP synthase D (ANN05233) | Dehydrated vs. Hydrated | 0.0088 | 1.172743 | UP |
|  | Rehydrated vs. Hydrated | 0.0478 | 1.12066 | UP |
| Nucleoplasmin (ANN11607; ANN11617) | Dehydrated vs. Rehydrated | 0.005 | 1.176306 | UP |
|  | Rehydrated vs. Hydrated | 0.0059 | 0.858287 | DOWN |
| Peptidase family C1 (ANN17613) | Rehydrated vs. Hydrated | 0.0227 | 1.178956 | UP |
| Peptidase M16 (AN19765; ANN05984) | Dehydrated vs. Rehydrated | 0.0094 | 0.859455 | DOWN |
|  | Rehydrated vs. Hydrated | 0.0412 | 1.118519 | UP |
| Protein of unknown function (ANN10400) | Dehydrated vs. Rehydrated | 0.0155 | 0.856115 | DOWN |
|  | Dehydrated vs. Hydrated | 0.0415 | 0.881481 | DOWN |
| Protein of unknown function (ANN10664; ANN01256) | Dehydrated vs. Rehydrated | 0.009 | 1.142973 | UP |
|  | Rehydrated vs. Hydrated | 0.0131 | 0.886888 | DOWN |
| Protein of unknown function (ANN11661) | Dehydrated vs. Hydrated | 0.0021 | 1.21365 | UP |
|  | Rehydrated vs. Hydrated | 0.0086 | 1.166172 | UP |
| Protein of unknown function (ANN14409) | Dehydrated vs. Rehydrated | 0.0012 | 0.852814 | DOWN |
|  | Dehydrated vs. Hydrated | 0.0012 | 0.840683 | DOWN |
| Ribophorin_II (ANN10919) | Dehydrated vs. Rehydrated | 0.011 | 1.135017 | UP |
|  | Dehydrated vs. Hydrated | 0.0021 | 1.198712 | UP |
| Sarcosine dehydrogenase (ANN04032) | Dehydrated vs. Rehydrated | 0.032 | 1.161227 | UP |
| Serpin (ANN13713; ANN11538) | Dehydrated vs. Rehydrated | 0.0082 | 0.865225 | DOWN |
|  | Dehydrated vs. Hydrated | <0.0001 | 0.790274 | DOWN |
|  | Rehydrated vs. Hydrated | 0.036 | 0.913374 | DOWN |
| Serpin (ANN13863; ANN27892) | Dehydrated vs. Rehydrated | 0.0167 | 0.836011 | DOWN |
| Thioredoxin (ANN17254) | Dehydrated vs. Hydrated | 0.006 | 0.790782 | DOWN |
|  | Rehydrated vs. Hydrated | 0.0394 | 0.854181 | DOWN |
| Trifunctional purine biosynthetic protein adenosine-3 (ANN12687) | Dehydrated vs. Rehydrated | 0.0071 | 0.816844 | DOWN |
|  | Rehydrated vs. Hydrated | 0.0071 | 1.247033 | UP |
